# Progress in Molecular Nanoarchitectonics and Materials Nanoarchitectonics

**DOI:** 10.3390/molecules26061621

**Published:** 2021-03-15

**Authors:** Katsuhiko Ariga

**Affiliations:** 1 WPI Research Center for Materials Nanoarchitectonics (MANA), National Institute for Materials Science (NIMS), 1-1 Namiki, Tsukuba, Ibaraki 305-0044, Japan; ARIGA.Katsuhiko@nims.go.jp; 2 Graduate School of Frontier Sciences, The University of Tokyo, 5-1-5 Kashiwanoha, Kashiwa, Chiba 277-8561, Japan

**Keywords:** bio-related application, energy-oriented application, nanoarchitectonics, nanotechnology

## Abstract

Although various synthetic methodologies including organic synthesis, polymer chemistry, and materials science are the main contributors to the production of functional materials, the importance of regulation of nanoscale structures for better performance has become clear with recent science and technology developments. Therefore, a new research paradigm to produce functional material systems from nanoscale units has to be created as an advancement of nanoscale science. This task is assigned to an emerging concept, nanoarchitectonics, which aims to produce functional materials and functional structures from nanoscale unit components. This can be done through combining nanotechnology with the other research fields such as organic chemistry, supramolecular chemistry, materials science, and bio-related science. In this review article, the basic-level of nanoarchitectonics is first presented with atom/molecular-level structure formations and conversions from molecular units to functional materials. Then, two typical application-oriented nanoarchitectonics efforts in energy-oriented applications and bio-related applications are discussed. Finally, future directions of the molecular and materials nanoarchitectonics concepts for advancement of functional nanomaterials are briefly discussed.

## 1. Introduction

Advancements of nanoscale science require the creation of a new methodology to produce functional materials systems from nanoscale units. As described in details later in this review manuscript, this task of advancements of nanoscale science is assigned to an emerging concept, nanoarchitectonics [1,2]. In this review article, various recent examples on applications of the nanoarchitectonics concept for production of functional materials are introduced, together with the basic processes of nanoarchitectonics. In this introductory section, the background and outline of the nanoarchitectonics concept are briefly described.

**Figure 1:**
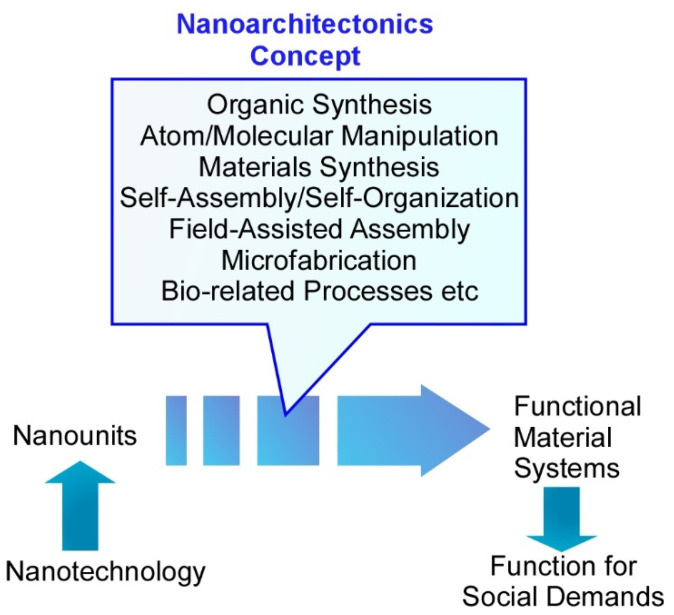
The nanoarchitectonics methodology to produce functional materials and functional structures from nanoscale unit components.

Production of functional materials has been traditionally pursued by various synthetic methodologies such as organic synthesis [3,4,5,6], polymer chemistry [7,8,9,10], and materials science [11,12,13,14]. Research developments in these synthetic processes revealed the importance of regulation of nanoscale structures within the corresponding materials for better performance and function. In parallel, analyses, observations, and manipulations of nanoscale objects have been scientifically and technologically developed in these decades. This progress created a new paradigm, nanotechnology [15,16,17,18], which was originated in the proposal by Richard Feynman [19,20,21] and reactivated in the late 20th century. Promoted understanding of nanoscale phenomena along with technological advancements in the manipulation and fabrications of ultrasmall objects opened huge possibilities for material fabrication with nanoscale structural information [22,23,24]. This scientific and technological progress also overlapped with developments in the other research fields such as supramolecular chemistry [25,26,27,28] and bio-related sciences [29,30,31,32] that deal with molecular organization. Based on these historical backgrounds, a novel conceptual paradigm next to nanotechnology was awaited to create unified methodology for production of functional material systems from nanoscale units. At the beginning of the 21st century, Masakazu Aono proposed a new paradigm, nanoarchitectonics, through combining nanotechnology with the other research fields such as organic chemistry, supramolecular chemistry, materials science, and bio-related science (Figure 1) [33,34].

The nanoarchitectonics methodology is supposed to produce functional materials and functional structures from nanoscale unit components through the combination and selection of various processes, including organic synthesis (especially heteromolecular synthesis), atom/molecular manipulation, materials synthesis, self-assembly/self-organization, field-assisted assembly, microfabrication, and bio-related processes [35,36]. Because these features can be applied to many kinds of materials, nanoarchitectonics strategies have been generally used for production of functional materials [37,38,39] and regulation of fine structures [40,41,42]. Not limited to material synthesis and fabrication, the nanoarchitectonics concept has been widely applied to various application-oriented fields such as catalysts [43,44,45], sensors [46,47,48], devices [49,50,51], energy-related applications [52,53,54], environmental applications [55,56,57], bio-related functions [58,59,60], and biomedical applications [61,62,63].

Unlike simple self-assembly processes based on equilibrated events, the nanoarchitectonics approaches are made by combinations and step-wise applications of various unit processes including energy-free equilibrium processes and energy-consuming non-equilibrium processes. Therefore, the nanoarchitectonics methods are advantageous for the fabrication of hierarchical structures [64]. This feature of nanoarchitectonics approaches is rather similar to the organization processes in biological systems upon certain energy consumption.

Since various uncertainties and fluctuations such as thermal fluctuations, statistical distributions, and quantum effects are not avoided among nanoscale material interactions, multiple interactions and processes have to be harmonized in the nanoarchitectonics processes rather than simple summation of unit processes [65]. The latter feature is similar again to those happening in biological systems where many functions work together in excellent harmony in a series of well-defined processes to produce complicated hierarchical structures even under non-negligible thermal fluctuations. Formation of functional materials systems by nanoarchitectonics approaches shares common features with the organization of biological systems.

Based on these backgrounds, basics and application examples of the nanoarchitectonics approaches are briefly explained in this review article. In the initial parts, basic-level nanoarchitectonics is mainly presented where atom/molecular-level structure formation and conversion from molecular units into functional materials are discussed. In the later sections, two typical application-featured nanoarchitectonics efforts in energy-oriented applications and bio-related applications are exemplified. The described examples cannot cover all the aspects but they provide the main common features in the nanoarchitectonics approach. Finally, future directions of the nanoarchitectonics concepts especially for advancement of nanoscale science are briefly discussed.

## 2. Basic Nanoarchitectonics

### 2.1. Atom/Molecular-Level Nanoarchitectonics, Observation

The smallest level of nanoarchitectonics events occurs at the atom/molecular scales [66]. Molecular-scale events such as chemical reactions and molecular associations have been investigated traditionally by various spectral methods through collecting average information of numerous molecules in solution. However, rapid developments of probe microscopies and electron microscopes enable us to directly observe individual molecules and their behaviour. Atom/molecular-level nanoarchitectonics can be evaluated on the basis of direct observations.

**Figure 2:**
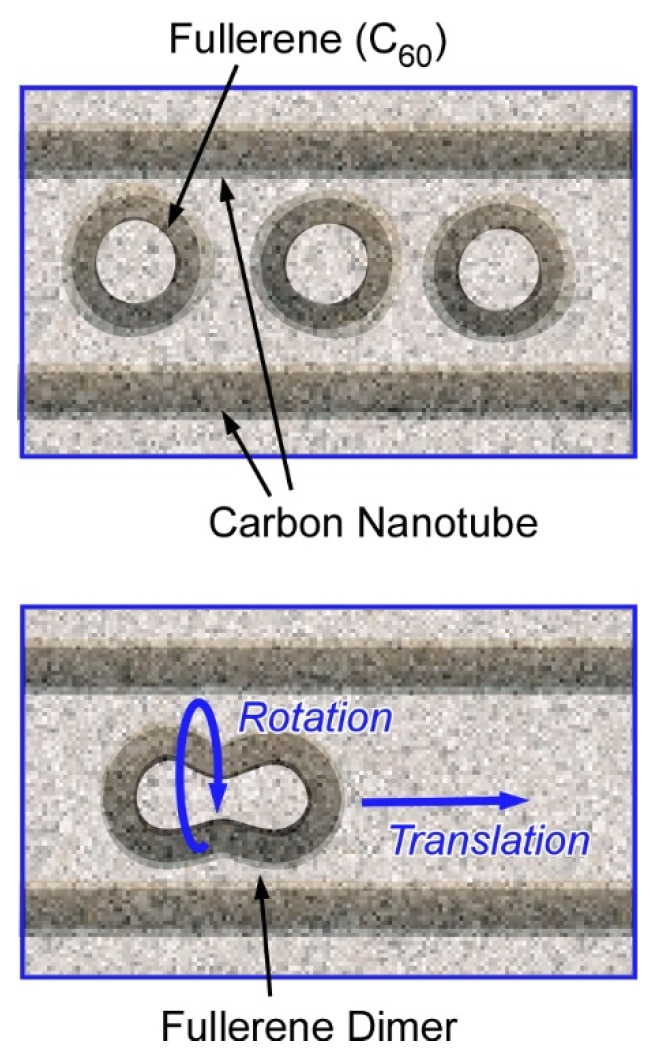
Nanoarchitectonics designs (models) of entrapment of fullerene molecules (C_60_ molecules) within a carbon tube for single-molecule atomic-resolution real-time electron microscopic (SMART-EM) with image recording.

Harano and co-authors have demonstrated various examples on observation of molecular behaviours with high spatial precisions and ultrashort resolutions using their technique, single-molecule atomic-resolution real-time electron microscopic (SMART-EM) with image recording [67,68]. For example, a single molecular level mechanical motions with sub-angstrom and sub-millisecond precision were recorded. Real-time recordings of nanoscale motions are realized using a fast camera with the aid of a denoising algorithm. Nanoarchitectonics design of entrapped fullerene molecules (C_60_ molecules) within a carbon tube revealed shuttling and rotating behaviours of a single C_60_ molecule (Figure 2) [69]. The molecular motions are coupled with carbon nanotube vibrations and can be observed in real-time mode with spatial resolution of 0.01 nm and standard error in time of 0.9 msec. The observed motions exhibited non-linear and stochastic natures and were often non-repeatable. This research revealed a molecular-level relationship between work and energy that had not been detected previously with time-averaged measurements and microscopic observations. In the used nanoarchitectonics motif, the carbon nanotube container and the entrapped C_60_ molecules behaved together as a mechanical coupled oscillator to show characteristics of chaotic systems. These observations would explain the infrequent and stochastic motional behaviours of molecules attached to a carbon nanotube.

**Figure 3:**
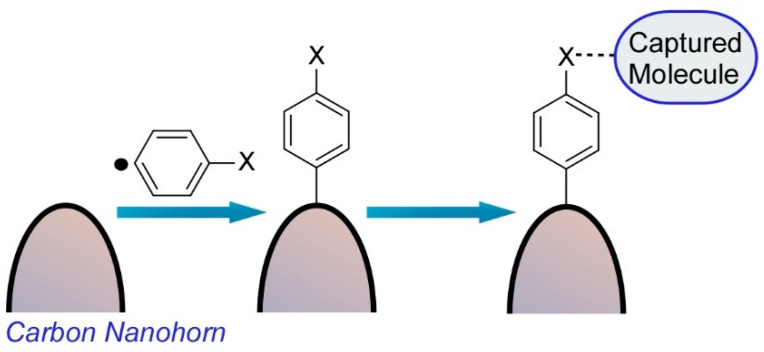
Molecular attachment to a surface of a carbon nanohorn for a single molecular level observation.

Harano and co-workers also reported a single molecular level observation of molecular attachment to a surface of a carbon nanohorn (Figure 3) [70]. This chemical fishhook could capture a single molecule from its solution and transfer the captured molecule into the nm-scale view field of the electron microscope, which are essential processes in the SMART-EM technique. As the initial stem of the chemical fishhook, an aromatic group was installed on the surface of carbon nanohorns through the selective attachment reaction of in-situ-generated aryl radicals from arylamines to strained parts of the graphitic surface with negative and positive curvatures.

The other molecular moieties can be further attached through amide bond formation and/or be assembled upon van der Waals interaction from their solution. The aryl group was reacted perpendicular to the graphitic carbon nanohorn surface and otherwise physisorbed on the surface. Characteristics of a biradical resonance between two bowl-like strained pentagon moieties connected by aromatic linker were expected, but monoradical addition was only observed on the most strained apex of the carbon nanohorn. The second radical site may accept a hydrogen atom from the solvent used.

**Figure 4:**
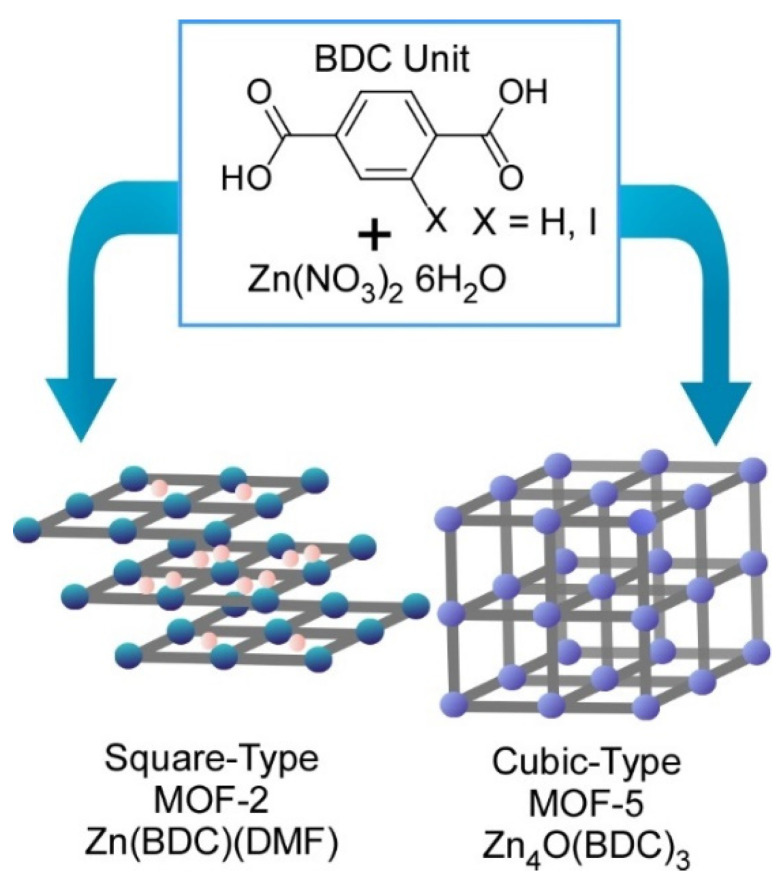
Two MOF structures (MOF-2 and MOF-5) obtained from the same precursors, zinc nitrate and benzene dicarboxylic acid in dimethylformamide under different conditions.

Fundamentals of coordination processes can be investigated with this technique. Atomic-level structure analyses on prenucleation clusters in syntheses of metal-organic frameworks (MOFs) were carried out using the SMART-EM technique by Harano and co-workers (Figure 4) [71]. As representative examples, two MOF structures (MOF-2 and MOF-5) can be obtained from the same precursors—zinc nitrate and benzene dicarboxylic acid—in dimethylformamide under different conditions.

This research revealed processes to differentiate these two MOFs formation at resolution level at a single prenucleation cluster. Two different types of the prenucleation clusters were detected in the formation processes for MOF-2 and MOF-5 at 95 °C and 120 °C, respectively. Formation of a small amount of 1-nm-size cube and cube-like prenucleation clusters was identified during MOF-5 synthesis processes. These prenucleation clusters were in turn not detected in the MOF-2 formation process where linear and square prenucleation clusters were only found. Bifurcation between MOF-2 and MOF-5 was initiated even at the atomic structure level of prenucleation clusters. These basic structure features are nanoarchitected into crystal-level morphologies with square MOF-2 and the cubic MOF-5 lattices. Initiation of nanoarchitectonics process from metal ions and ligands to macroscopic MOF materials can be visualized by forefront microscopic techniques in the current technology.

### 2.2. Atom/Molecular-Level Nanoarchitectonics, Synthesis

**Figure 5:**
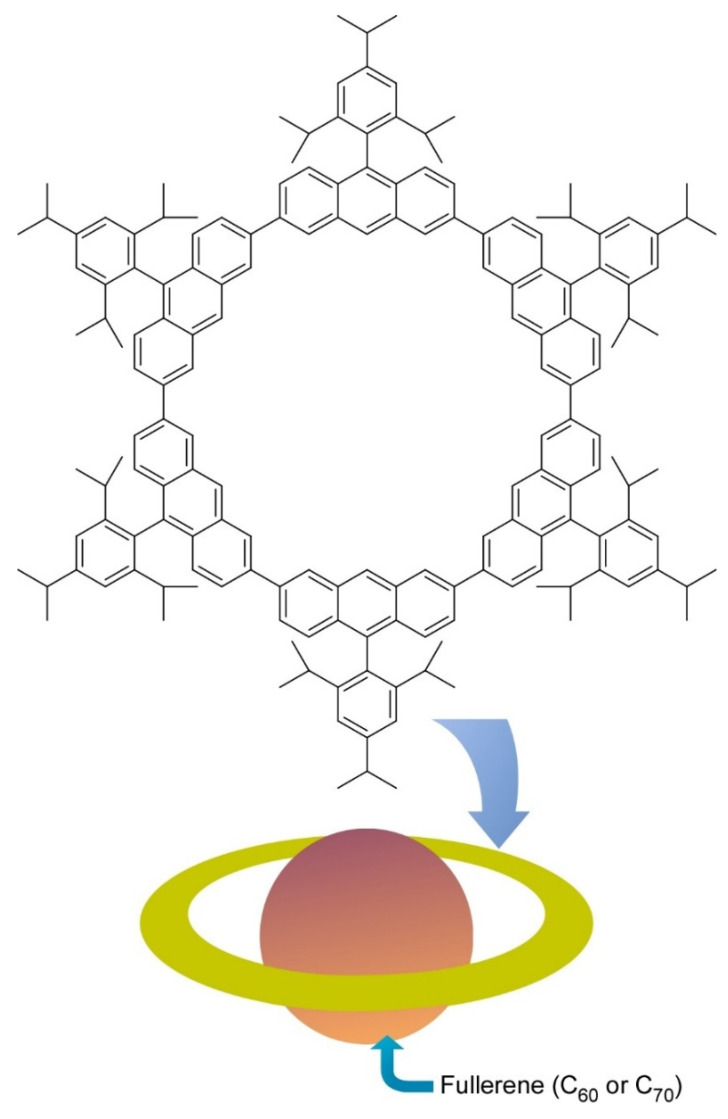
Nano-Saturn through supramolecular association between anthracene macrocyclic ring and ellipsoidal C_70_ molecule.

Molecular level nanoarchitectonics are driven by non-covalent molecular associations and/or covalent organic reactions. Some innovative molecular-level nanoarchitectonics approaches from nanocarbon-related molecular nanoarchitectonics are exemplified below. As an example of the molecular association approach, Toyota et al. reported successful preparation of a nano-Saturn structure through supramolecular association between anthracene macrocyclic ring and ellipsoidal C_70_ molecule (Figure 5) [72]. Unlike typical supramolecular complex such as alkali ion trap by crown ethers, weaker CH-π interactions have significant contributions in this ring-body supramolecular complex. Association constant of C_70_ molecules to the anthracene macrocyclic ring was twice larger than that for complex formation with C_60_ molecule. the central fullerene guest can float from the center of the ring without causing serious deterioration of their binding constant. This nanoarchitectonics motif is advantageous to form supramolecular complexes with non-spherical fullerenes.

**Figure 6:**
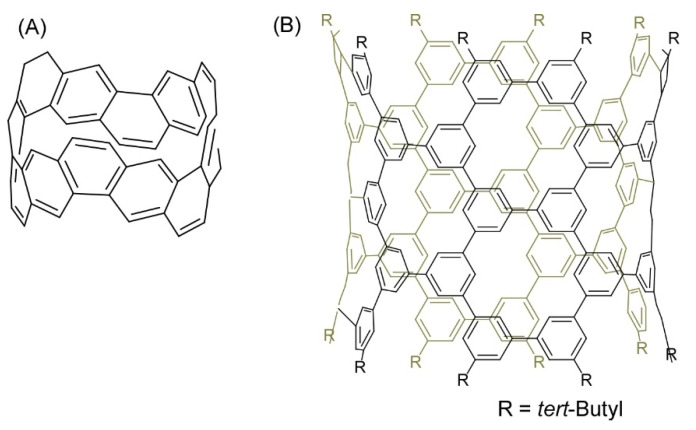
(**A**) Carbon nanobelt with a closed loop of fully fused edge-sharing benzene rings; (**B**) Cylindrical C_304_H_264_ molecule with 40 benzene (phenine) units bonded mutually at the 1, 3, and 5 positions.

Several recent examples have demonstrated the contributions of skilled organic synthesis approaches to nanoarchitectonics to produce nanocarbon materials. Segawa and co-workers successfully demonstrated the synthesis of a carbon nanobelt, which is a closed loop of fully fused edge-sharing benzene rings (Figure 6A) [73]. The carbon nanobelt was synthesized through iterative Wittig reactions that were followed by a nickel-mediated aryl-aryl coupling reaction. It is expected to further nanoarchitect carbon nanotube materials with well-defined structures using the carbon nanobelt molecules as seed units. Carbon nanotube materials with uniform diameter and single chirality would be produced upon programmed synthesis with carbon nanobelt derivatives. Sun, and co-workers also demonstrated the synthesis of cylindrical C_304_H_264_ molecules with 40 benzene (phenine) units bonded mutually at the 1, 3, and 5 positions as a finite phenine nanotube with periodic vacancy defects (Figure 6B) [74]. Nanoarchitecting carbon nanotubes with electronic properties modulatable by periodic vacancy defects upon fusion of the synthesized cylinder molecules were suggested by computational approaches.

**Figure 7:**
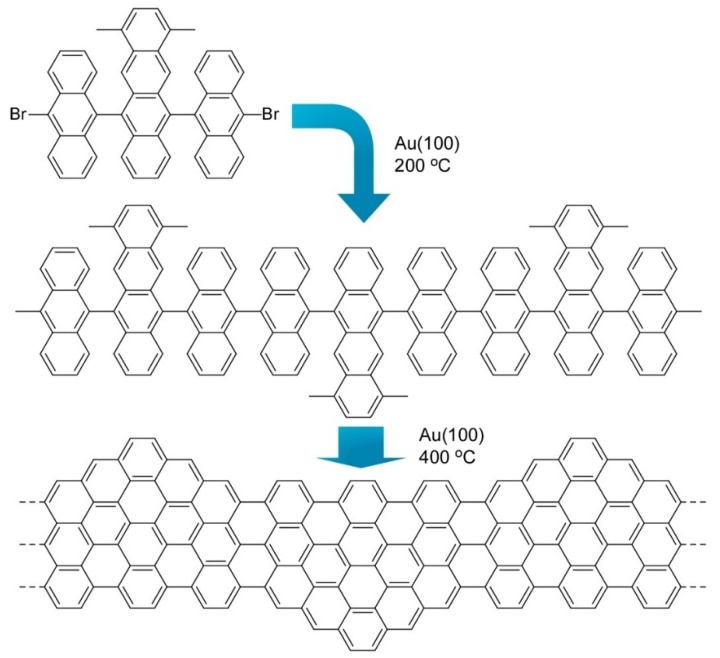
Fusion of molecules with a dimethyltetracene core and two bromoanthryl units into structure defined graphene nanoribbons.

Nanoarchitectonics from organic molecules to two-dimensional nanocarbon has been also investigated as exemplified in a recent review article by Xu et al. [75,76,77]. Synthesis of the structure-defined graphene nanoribbons was accomplished through fusion of discrete polycyclic aromatic hydrocarbons at a solid surface. Therefore, this type of synthetic approach is often called on-surface synthesis. Figure 7 shows one example where precursor molecules with a dimethyltetracene core and two bromoanthryl units were fused into structure-defined graphene nanoribbons. Precisely prepared graphene nanostructures including graphene nanoribbons and graphene quantum dots are capable of having open bandgaps because of their quantum confinement effect. This characteristic is much different from zero-bandgap graphene and is attractive for semiconductor-related applications such as optoelectronics and nanoelectronics. However, preparation of precisely structurally controlled graphene nanostructures is difficult with conventional material processing. Bottom-up nanoarchitectonics from molecular precursors to well-defined nanocarbon with on surface synthesis would open many possibilities of nanocarbon technology.

**Figure 8:**
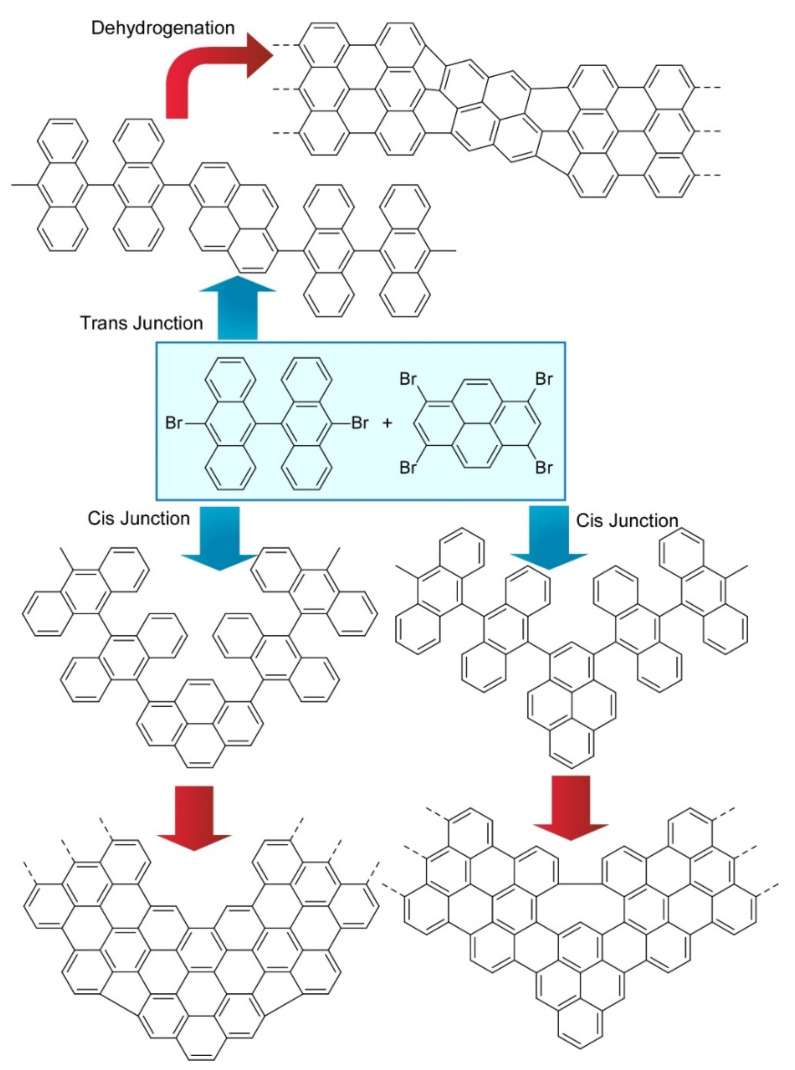
Three different regioisomeric junctions synthesized from 10,10’-dibromo-9,9’-bianthryl and 1,3,6,8-tetrabromopyrene on Au (111) surface.

**Figure 9:**
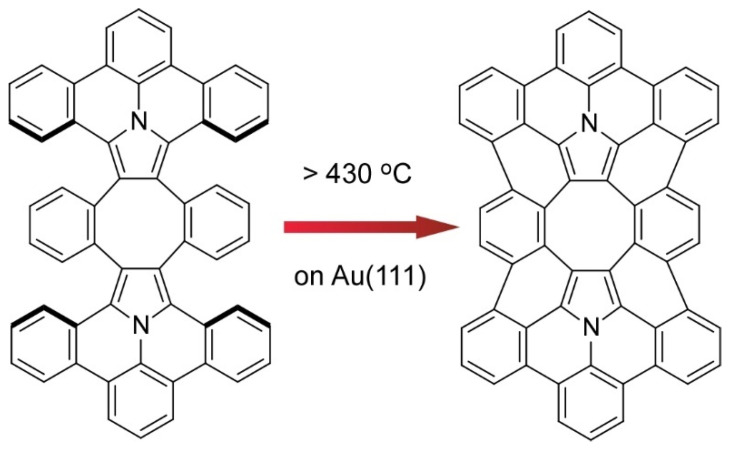
Synthesis of π-extended diaza[8]circulene through on-surface syntheses on a Au(111) surface.

Kawai and co-workers demonstrated on-surface synthesis for regioisomeric graphene nanoribbons through fusion of two kinds of precursor molecules (Figure 8) [78]. Three different regioisomeric junctions were synthesized from 10,10’-dibromo-9,9’-bianthryl and 1,3,6,8-tetrabromopyrene on a Au (111) surface. When a sufficient amount of 10,10’-dibromo-9,9’-bianthryl relative to 1,3,6,8-tetrabromopyrene was supplied, 10,10’-dibromo-9,9’-bianthryl molecules were reacted at bromo-substituted sites in 1,3,6,8-tetrabromopyrene through an Ullmann-type reaction. Depending on the geometric relation between two reaction sites, subsequent cyclodehydrogenation upon high-temperature annealing resulted in graphene nanoribbon junctions with different connecting angles. Further analyses by scanning tunnelling spectroscopy with a CO-terminated tip with the aid of density functional theory (DFT) calculations revealed chemical structures and the electronic properties of these structure-defined graphene nanoribbons. The demonstrated nanoarchitectonics strategy would be applied to the other units to produce various carbon nanostructures. Nakamura et al. reported the synthesis of π-extended diaza[8]circulene through a combination of in-solution and on-surface syntheses (Figure 9) [79]. The final form of π-extended diaza[8]circulene possessing six hexagons and two pentagons cannot be obtained only with solution-based reaction processes. The final cyclodehydrogenation step has to be done on a Au(111) surface.

**Figure 10:**
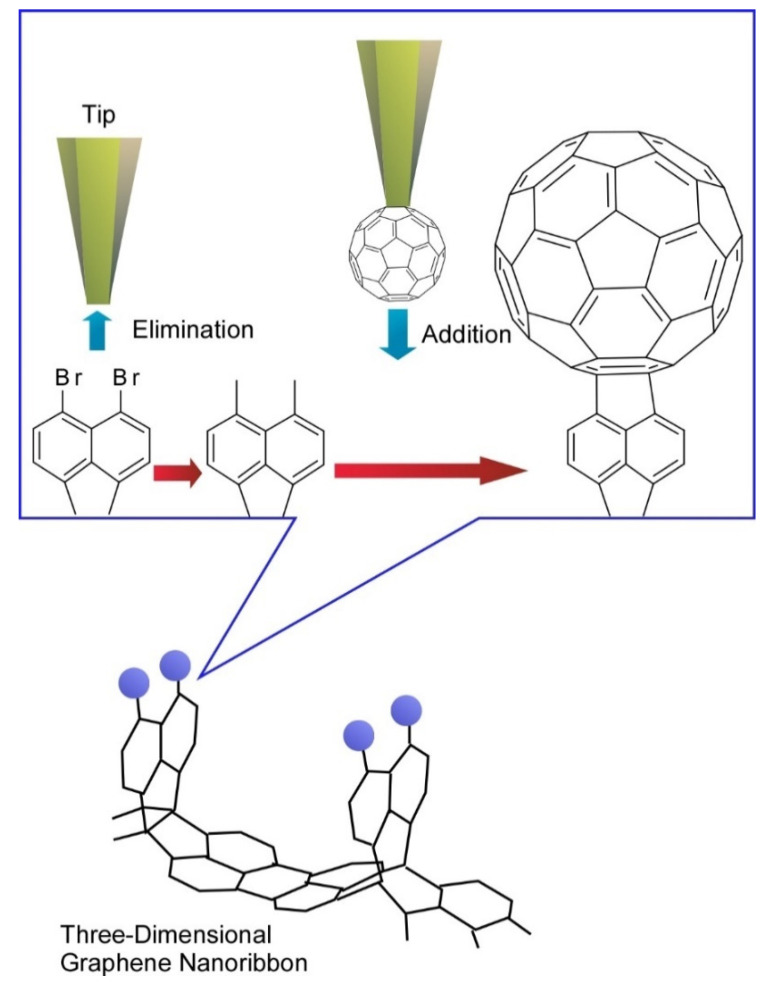
Tip-induced debromination with substitution reaction with fullerene molecule were at three-dimensional graphene nanoribbon.

In more advanced approaches to organic molecular nanoarchitectonics, tip-induced reactions have been investigated. Organic syntheses are mediated through molecular manipulations using the tip of probe microscopes that is called local probe chemistry. In a recent example reported by Kawai et al., three-dimensional graphene nanoribbons were first prepared by on-surface chemical reaction and tip-induced debromination with substitution reaction were demonstrated (Figure 10) [80]. The debromination process resulted in unstable radical species through cleaving the out-of-plane C-Br bond. The local probe chemistry was carried out at low temperature under ultra-high vacuum, which stabilized unstable debrominated radical species. Subsequently, a fullerene C_60_ molecule attached to the tip apex of the probe was directly transferred to the reactive radical site on graphene nanoribbon to complete substitution reactions. This example implies that nanoscale science would play important roles even in organic chemistry, which would enable us to synthesize target molecules through even atom-by-atom nanoarchitectonics.

**Figure 11:**
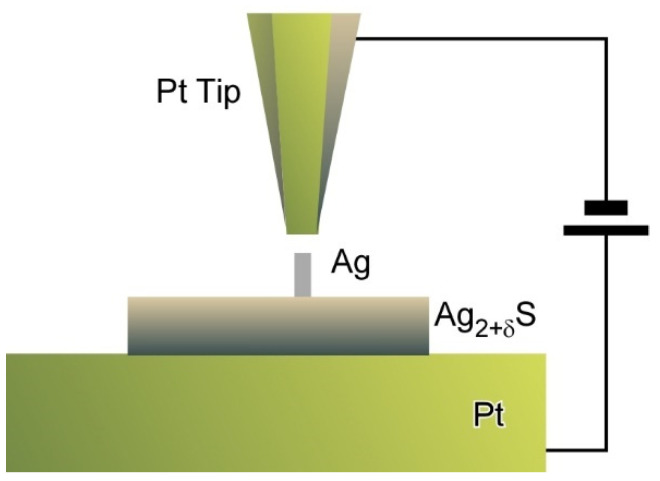
Nanoarchitectonics strategy to control the numbers of dopant atoms within solid electrolyte nanostructures using a Pt tip.

Tip-mediated manipulations can be applicable to inorganic semiconductor materials as reported by Hasegawa and co-workers who proposed a nanoarchitectonics strategy to control the numbers of dopant atoms within solid electrolyte nanostructures (Figure 11) [81]. A Pt tip was positioned above α-Ag_2+σ_S nanodots as a model system with non-stoichiometry excess dopants at a tunnelling distance. Electrochemical precipitation of Ag atoms to form a Ag protrusion was initiated when the bias voltage was increased to 100 mV. Step heights of protrusion growth corresponded to multiples of single atomic plane of Ag (111) and finally reached to equilibrated height at the given bias. These stepwise precipitations of Ag resulted in tuning of the numbers of excess dopants at an atomic level. As the results, atom-by-atom-level tuning of electrochemical potential energy can be achieved. The proposed nanoarchitectonics approach to manipulate the numbers of dopant atoms in solid electrolyte materials upon control of applied bias leads to discrete regulation of electrical properties of nanomaterials. Eventually, this could become a promising method to develop nanomaterial devices with single ion/atom transfer capability.

**Figure 12:**
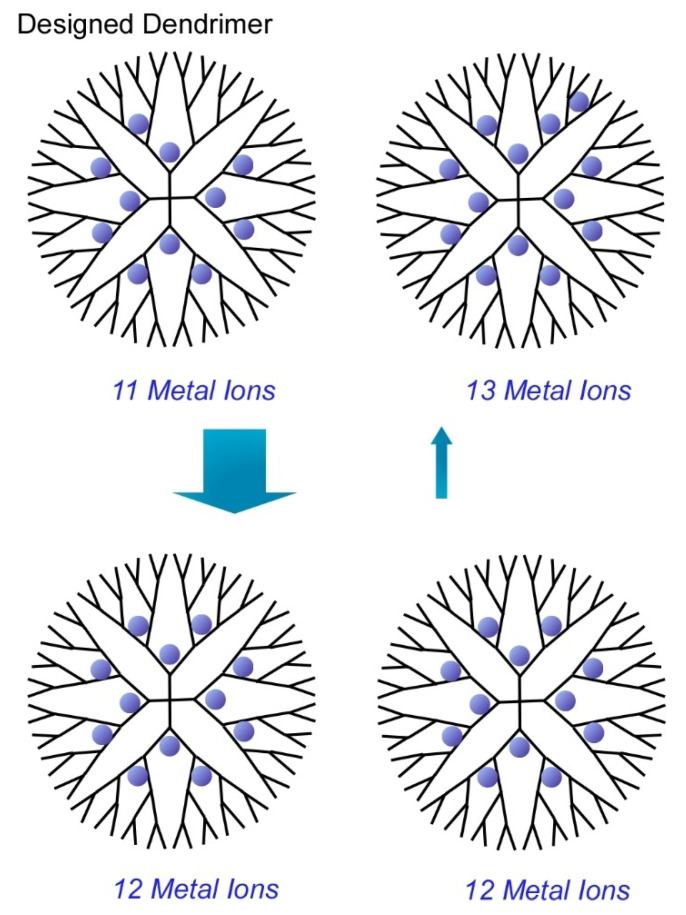
Structurally defined dendrimer cores to give metal cluster with precisely controlled number (12 atoms).

Another target of atomic-level precise nanoarchitectonics would be synthesis of metal clusters with discrete numbers of atoms. Ultimately, functional metal clusters are desirably prepared in ultraprecise control of their size at a single atom level. In a recent review article by Imaoka and Yamamoto [82,83,84], chemical approaches to synthesize atomically precise metal clusters are discussed. Their strategies basically utilized basicity gradient within structurally defined dendrimers to which metal ions can be coordinated. In the case of the dendrimer template depicted in Figure 12, 12 metal ions can be complexed at the coordination sites up to the dendrimer second layer and 28 atoms can coordinate up to the third layer. Based on the clear differences of the basicity of coordination sites between the second and third layers, discrete numbers of metal ions were isolated within the dendrimer cores to give metal cluster with precisely controlled number (12 atoms). For example, synthesis with use of phenylazomethine-based dendrimer template provided atomically controlled Pt clusters on the basis of sufficient basicity gradient strength of the dendrimer template.

These examples demonstrate various types of atom/molecular-level nanoarchitectonics to create functional structures and materials from atomic and molecular structural unites. In addition to chemical techniques and surface sciences, nanotechnological tools such as tips of probe microscopies are used in advanced examples. Although organic syntheses are recognized as well-established research fields, advanced nanoscale technique can open new pages even in this classic science. This would be a successful nanoarchitectonics example of field fusion between nanotechnology and traditional science.

### 2.3. Nanoarchitectonics toward Materials

In order to prepare functional systems useful in many occasions, conversion from molecular units (or nanomaterial units) to functional materials is a crucial process. In such conversions, reflection of structural and functional features of nanounits is important to keep the high functions in material level. These nanoarchitectonics processes from nano to materials have potential contributions to many research fields including supramolecular chemistry [85,86,87,88] and materials chemistry [89,90,91,92] although they were not recognized as parts of nanoarchitectonics. However, many of them bear features of material-level nanoarchitectonics. For preparation for nanofeature-bearing functional materials, various assistant factors such as guiding by template structures and asymmetrical structure formation at interfacial environments have important roles in addition to conventional self-assembly.

**Figure 13:**
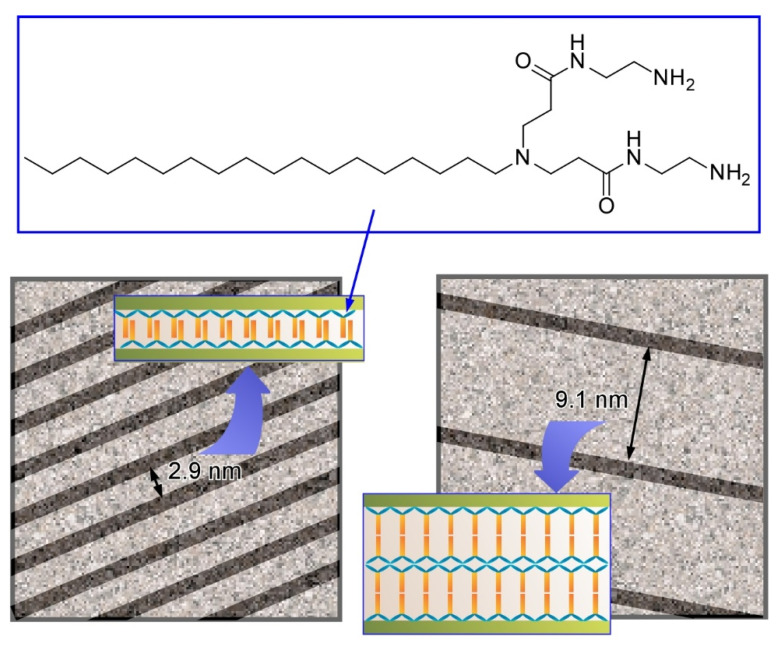
Parallel allays of the Au nanowires with regular wire-by-wire intervals (models) with narrow interval of 2.9 nm and wide interval of 9.1 nm, corresponding to interdigitated bilayer and non-interdigitated four layer of the ascorbic acid derivatives, respectively.

For example, Kawai and co-workers successfully synthesized ultrathin Au nanowires in aqueous systems with guiding of molecular assemblies of ascorbic acid derivatives, and subsequent alignment of the synthesized Au nanowires with precise intervals was demonstrated (Figure 13) [93]. Au nanowires with a diameter of ca. 1.7 nm were fabricated through an oriented attachment growth mechanism. Ascorbic acid derivatives with octadecyl chains weakly attached on the Au(111) crystal face induced oriented growth of the Au nanowire. Elongation of the nanowires was effectively facilitated in the presence of Cl^−^ ions to give nanowires with a length of over a few µm. Drying processes of the aqueous Au nanowire solutions on a solid substrate resulted in parallel allays of the Au nanowires with regular wire-by-wire intervals. Mainly narrow intervals of 2.9 nm and wide intervals of 9.1 nm were observed. The former intervals (2.9 nm) correspond to the bthickness of the interdigitated bilayer of the ascorbic acid derivatives. Formation of a non-interdigitated four layer (double bilayer) between the Au nanowires can explain the wide interval (9.1 nm). These examples demonstrate that simple amphiphile assemblies can guide the formation of micro-level structures with sub-nanometer-level internal structural precision.

A similar guiding method can be applied to other material systems. As summarized an extensive recent review article by Akagi [94,95], chiral conjugate polymer materials with the guide of chiral liquid crystalline templates. Helical screw directions (materials chirality) can be selected by the chiral dopants in liquid crystals. Controlled helical structures of conjugated polymers was led to chiroptical properties such as circularly polarized luminescence. Furthermore, helical conjugate polymer materials can be converted into graphitic carbons without causing structural deteriorations of the original helical structures by iodine-doped carbonization.

**Figure 14:**
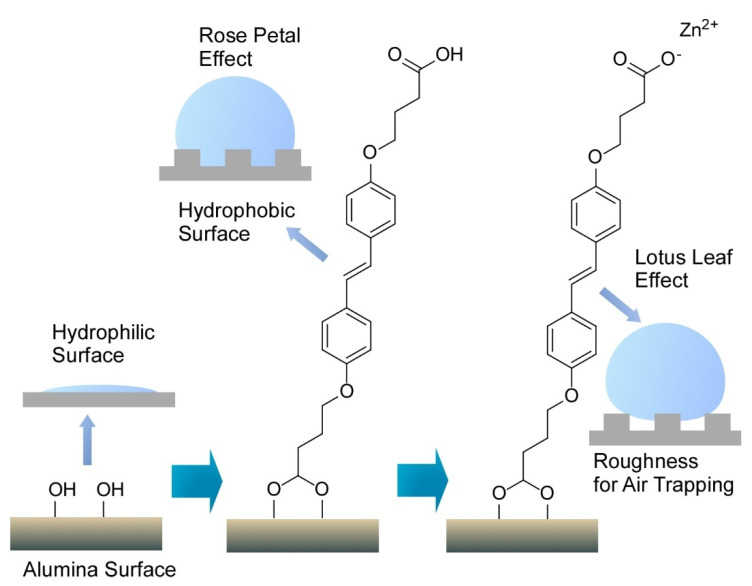
Surface nanoarchitectonics of conventional alumina materials to regenerate bio-like wettability functions of rose petal and lotus leaf effects where single molecule in assembled structure is only depicted.

Interfaces are nice playgrounds to produce various functional material properties [96]. Nanoarchitectonics at interfaces is advantageous for delicate tuning of functions [97]. For example, Ajayaghosh and co-workers delicately nanoarchitected the surface of conventional alumina materials to regenerate the bio-like wettability functions of rose petal and lotus leaf effects (Figure 14) [98]. The former effect induces sticky water droplets through droplets pinned on surface nanostructures, and slippery water droplets are observed with the latter effect with droplet sitting on the top surface of the nanostructures. Intrinsically hydrophilic aluminum surface was first modified with (*E*)-4,4’-(diazene-1,2-diyl)bis(4,1-phenylene))bis(oxy)dibutanoic acid to give a water contact angle of 145° with high contact angle hysteresis of ±69° advantageous for water sticking. Further coordination with Zn^2+^ ions resulted in a higher contact angle to water (165°) and lower contact angle hysteresis (±2°) for water slipping. In both the cases, coating with an aromatic bis-aldehyde with alkoxy chain substituents were required to express rose petal and lotus leaf effects. This adduct worked as nanowaxy cuticle in naturally occurring systems. Surface nanoarchitectonics with light tuning of modification and coating structures can convert conventional alumina materials into bio-like functional surfaces.

Liquid surfaces such as gas-liquid interfaces and liquid-liquid interfaces have several advantages for nanoarchitectonics processes from molecular/nanomaterial level to functional materials. Interfacial environments between two immiscible liquids would give encountering opportunities for molecular components with different solvent affinities. These situations are well suited to nanoarchitect two-dimensional metal-organic frameworks (MOFs) [99,100,101] and covalent organic frameworks (COFs) [102,103,104]. Drastic changes of component solubilities at liquid-liquid interfaces are used for materials nanoarchitectonics through liquid-liquid interfacial precipitation. For example, upon the liquid-liquid interfacial precipitation, fullerene molecules (C_60_, C_70_ and so on) can be nanoarchitected into various nano and microstructures [105,106,107] including one-dimensional rods/tubes/whiskers [108,109], two-dimensional sheets [110,111], three-dimensional cubes [112], hierarchical structures such as rod-on-cube [113,114] and hole-in-cube [115], and the other integrated structures [116,117,118].

### 2.4. Langmuir-Blodgett Nanoarchitectonics

**Figure 15:**
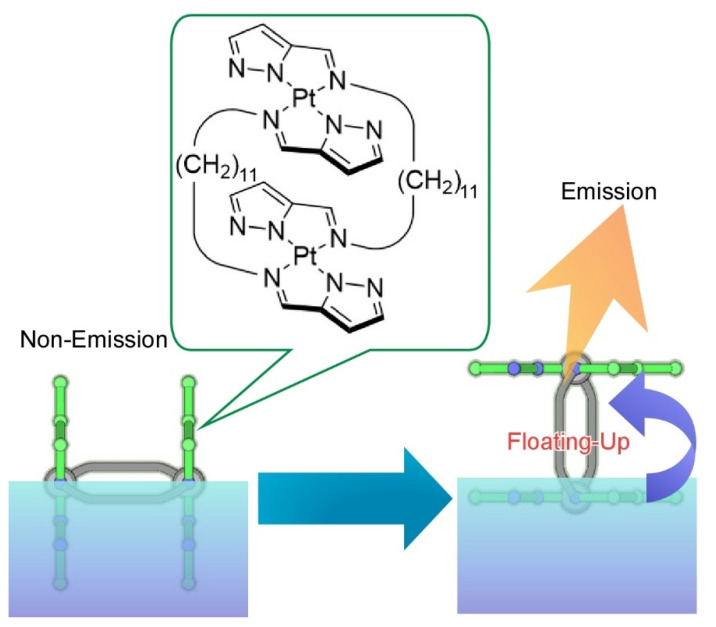
Faint orientational changes of double-paddled binuclear Pt^II^ at the air-water interface from perpendicular to parallel accompanied with drastic increase of phosphorescence, as called submarine emission.

As one of the typical thin film nanoarchitectonics methods, the Langmuir-Blodgett (LB) technique [119,120,121,122] is basically used at the air-water interface, where molecular recognition capabilities are drastically enhanced as compared with bulk aqueous phase [121,122], which has been demonstrated experimentally [123,124,125,126], spectroscopically [127,128,129,130], and theoretically [131,132]. This nature can be used for preparation of two-dimensional molecular patterns which have macroscopic lateral dimensions and sub-nanometer-level internal pattern structures [133]. Highly anisotropic motional freedoms at the air-water interface enable us to manipulate molecules by macroscopic motion like hand motion [134,135]. Macroscopic motions such as sub-meter-level compression and expansion of Langmuir monolayer in lateral direction can be coupled with molecular-level functions within nanometer-level thickness at the air-water interface. Regulation of molecular machines [136] by hand-like macroscopic mechanical motions such as reversible guest capture [137,138], enantio-selective amino acid discrimination [139], faint tuning of nucleic acid base recognition [140], control of fluorescence resonance energy transfer [141], molecular rotor rotation [142,143], molecular pliers operation [144,145], molecular flapping [146], and nanocar actions [147] have been actually demonstrated. As depicted in Figure 15, faint orientational changes of double-paddled binuclear Pt^II^ complexes through macroscopic mechanical compression of their monolayer at the air-water interface [148]. Molecular-level orientation change of the binuclear Pt^II^ complexes into chromophore emergence from water through molecular flapping from perpendicular to parallel was successfully induced accompanied with a drastic increase of phosphorescence. This emission increase by floating-up molecules from aqueous phase is called submarine emission.

**Figure 16:**
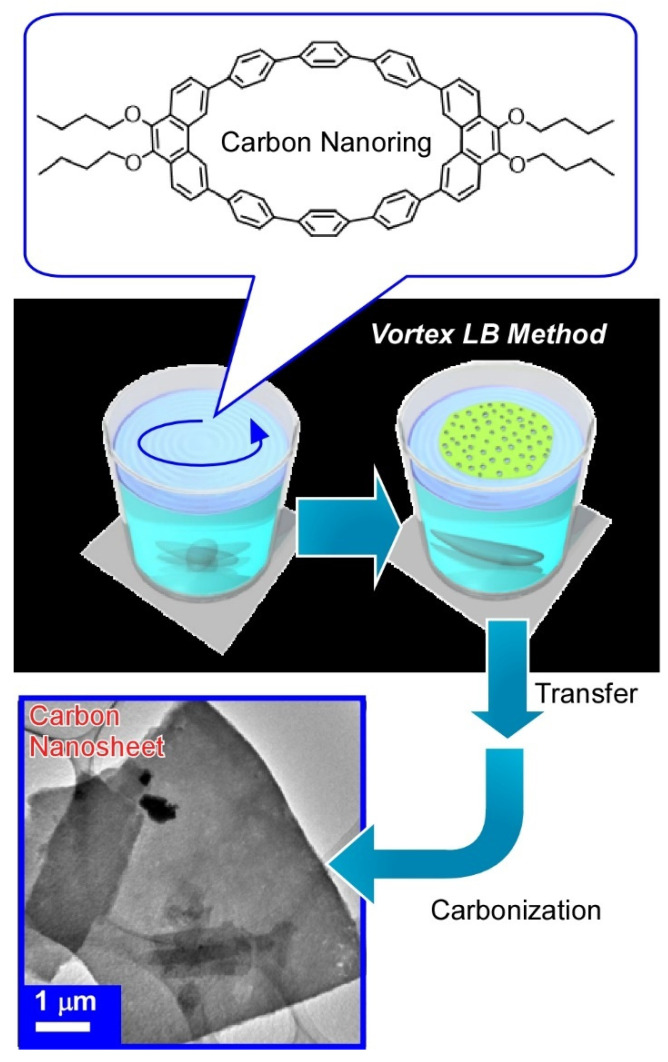
Nanoarchitectonics for preparation of carbon nanosheet from carbon ring molecule (9,9’,10,10’-tetrabutoxy-cyclo-[6]-paraphenylene-[2]-3,6-phenanthrenylene) through vortex Langmuir-Blodgett (vortex LB) method and calcination at 850 °C under N_2_ gas flow.

With dynamic process at the air-water interface, molecular precursors can be converted into structure-controlled nanomaterials as exemplified in Figure 16 [149]. In this case, carbon ring molecule (9,9’,10,10’-tetrabutoxycyclo-[6]-paraphenylene-[2]-3,6-phenanthrenylene) was selected as a molecular precursor. A molecular film of the carbon ring molecule was first spread through dropping its chloroform solution onto a water with a vortex rotating motion. This is called the vortex Langmuir-Blodgett (vortex LB) method [150]. Two-dimensional uniform thin films of carbon ring molecules were formed with the aid of vortex motion of the water phase. Analyses on the transferred film from the water surface onto a solid substrate revealed a uniform ultrathin nature (thickness of ca. 10 nm and width of tens of micrometers) and insulative properties. Calcination of the transferred film at 850 °C for 3 h under a N_2_ gas flow successfully converted the assembled film from a nanocarbon film without any structural deterioration accompanied with drastic increase of electrical conductivity (1.98 × 10^3^ Sm^−1^). Addition of pyridine during the initial vortex LB process efficiently resulted in nitrogen-doped carbon nanosheets with further increase of conductivity. Easy nanoarchitectonics methods from simple molecules into nitrogen-doped carbon nanosheet would become useful for preparation of efficient catalysts for oxygen reduction reactions in fuel cell applications.

In nanoarchitectonics processes from molecules to materials, regulation of molecular orientations within the nanoarchitected materials becomes key one of the important key factor for functions. As described in a recent review article by Kido and co-workers [151,152,153], molecular orientation is an indispensable factor to achieve high performances in organic light-emitting devices. They even expect that molecular engineering to nanoarchitect horizontal molecular orientation would open a golden era of vibrant research for organic light-emitting devices. Therefore, nanoarchitectonics methods to achieve well-controlled molecular orientation in materials such as ultrathin films become crucially important. Some established techniques such as the LB method [154,155] and layer-by-layer (LbL) assembly [156,157,158] have been applied to this task. However, fabrication of functional molecules and polymers into well-organized high quality thin films is not always easy, unlike conventional assembly of lipid molecules. Functional molecules with conjugated aromatic cores tend to form undesirable aggregates even in these conventional fabrication processes for ultrathin films.

**Figure 17:**
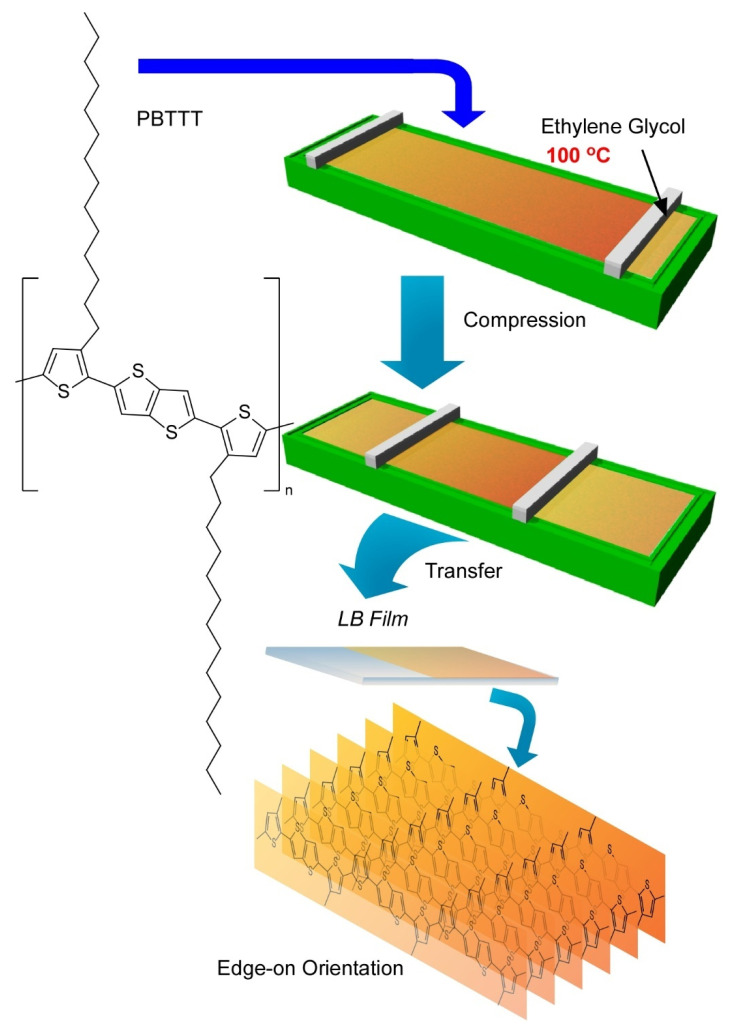
A novel method, 100 °C-Langmuir-Blodgett (100-LB) technique to fabricate highly oriented uniform ultrathin films with edge-on orientation of polymeric semiconductors, poly[2,5-bis(3-tetradecylthiophen-2-yl)thieno(3,2-b)-thiophene] (PBTTT), on ethylene glycol as a solvent for subphase.

Very recently, Ito et al. have demonstrated a breakthrough method, the 100 °C-Langmuir-Blodgett (100-LB) method, to fabricate highly oriented uniform ultrathin films of polymeric semiconductors (Figure 17) [159]. In common sense of science and technology, a conventional LB method is conducted at around room temperature. Because of unavoidable disturbances by vapours of water as a subphase liquid, LB processes above 40 °C are usually unfavourable. This limitation of operational temperature ranges is not advantageous to suppress undesirable aggregations of aromatic conjugate molecules. Ito et al. used ethylene glycol as a solvent for the subphase instead of water. The liquid range of ethylene glycol (−12.9 to 197.3 °C) led to a wide operational temperature for the LB technique where relatively low vapor pressures and high surface tensions can be maintained. Actually, LB film preparation was demonstrated up to 100 °C using a polymeric semiconductor molecule, poly[2,5-bis(3-tetradecylthiophen-2-yl)thieno(3,2-b)-thiophene] (PBTTT), which is known as a highly aggregative polymers with low solubility.

Thin films of this polymeric semiconductors were prepared through Langmuir-Schaefer-type transfer of surface films that were compressed after spreading at various temperatures up to 100 °C. LB films with defined thickness with high homogeneity over millimeter scales were obtained. Observation of the film surface morphology by laser confocal microscopy verified this high film homogeneity. High contrast in polarized optical microscopy images with different polarization angle implied significant orientation of the film where the main chains of polymeric semiconductor was highly oriented at least in a length scale of several hundred micrometer. Further analyses of the LB films with grazing incidence X-ray diffractions and grazing incidence wide-angle X-ray scattering revealed uniaxially aligned highly crystalline nature with edge-on lamellar orientation that is desirable for facilitated charge transport. The degree of crystallinity and alignment the LB films of the polymeric semiconductor tended to be enhanced with the increase of process temperature. The mobilities along the direction parallel to main polymer chains for the LB films prepared at 80 °C were obtained as high as 0.54 cm^2^V^−1^s^−1^. The obtained values are much higher than those observed for conventional thin films and that for room-temperature prepared LB film (0.17 cm^2^V^−1^s^−1^). The mobility parallel to main chains of the polymeric semiconductor in LB films prepared at 80 °C was eight times higher than that measured for the perpendicular direction to the main chains in the same LB film. These facts clearly proves the excellent performances of the polymeric semiconductor films based on higher degree of crystallinity and unidirectional properties through nanoarchitectonics of the high temperature LB technique.

As mentioned above, nanoarchitectonics from molecules to materials can produce various possibilities of functional materials with inside nano-organized structure. Interfacial processes that often play important roles in the fabrication of materials with nanostructure-based functions [160,161].

## 3. Advanced Nanoarchitectonics Applications

Fabrication of fine structures is important for the production of functional materials with high efficiency and high specificity. Nanoarchitectonics approaches to fabricate functional materials from nanoscale units are promising strategies. Even though the term of nanoarchitectonics is not directly mentioned, features and essences of the nanoarchitectonics are widely included in many examples to produce functional material systems. In the following sessions, several examples of functional material systems fabricated with essences of nanoarchitectonics concept are introduced mainly for two major practical fields, energy-oriented applications and bio-related applications.

### 3.1. Energy-Oriented Applications

Energy-related applications to produce energy and manage energy are undoubtedly socially important issues in current science and technology [162,163,164] as well as environmental problems [165,166,167] accompanied with sensing [168,169,170] and remediation technologies [171,172,173]. Electrochemical and electrical charge storages and energy conversions with various catalysts including chemical catalysts [174,175,176], electrochemical catalysts [177,178,179], and photocatalysts [180,181,182] have important roles in the corresponding functions. In the most of approaches for these research targets, structural constructions with nanoscale components (nanoarchitectonics) are actually investigated to get better performances [183,184].

**Figure 18:**
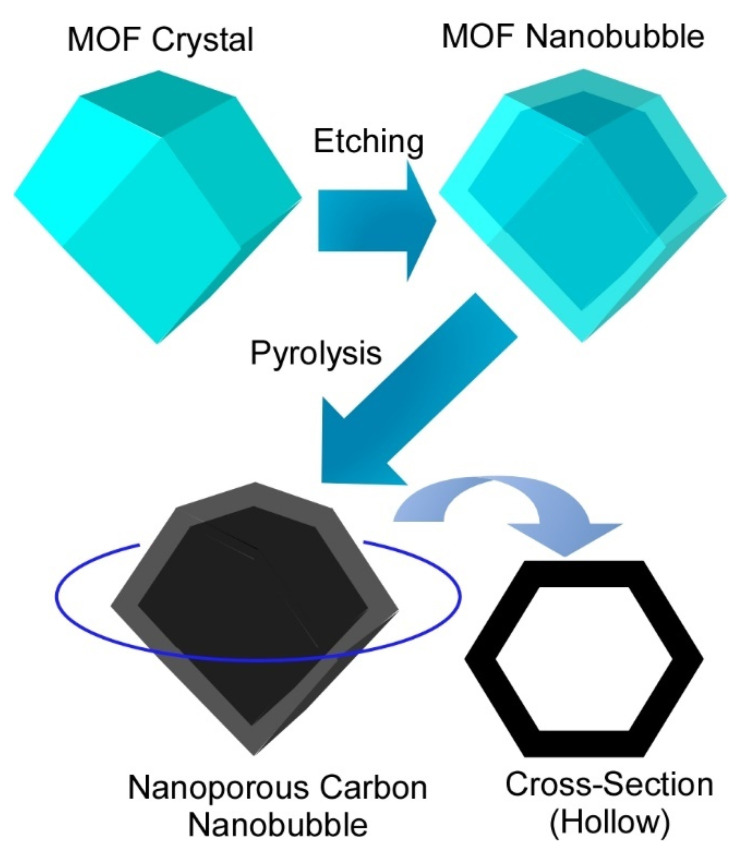
Nanoarchitectonics approach to fabricate hollow MOF nanobubbles and their carbonized materials by combined processes of MOF coordination self-assembly, site-selective etching, and calcination.

For example, Yamauchi and co-workers proposed a nanoarchitectonics approach to fabricate hollow nanobubbles with monocrystalline shells of MOFs and their carbonized materials by combined processes of MOF coordination self-assembly, site-selective etching, and calcination (Figure 18) [185]. The mother MOF structure, zeolitic imidazolate framework (ZIF-8), was first synthesized and further etched into nanobubble structures. During the etching process, protons for etching diffused into central core region of ZIF-8 through pores and nanochannels, resulting in selective core-etching with nanoscale structural precision. The outer region on ZIF-8 remained intact to give monocrystalline shell framework structures. The nanoarchitected materials possessed a uniform size of less than 100 nm and 10-nm-thick monocrystalline shells. The hollow ZIF-8 nanobubbles were further converted into nanoporous carbon nanobubbles through pyrolysis without causing any structural deteriorations. The fabricated structures exhibited enhanced performance of fast Na^+^/K^+^ ion intercalation as capacitor-type intercalation behaviours. Because conventional MOFs and related carbon materials cannot show similar superior performances, the proposed nanobubble nanoarchitectonics opens a new avenue of nanoshell-dependent electrochemistry for superior battery performance.

**Figure 19:**
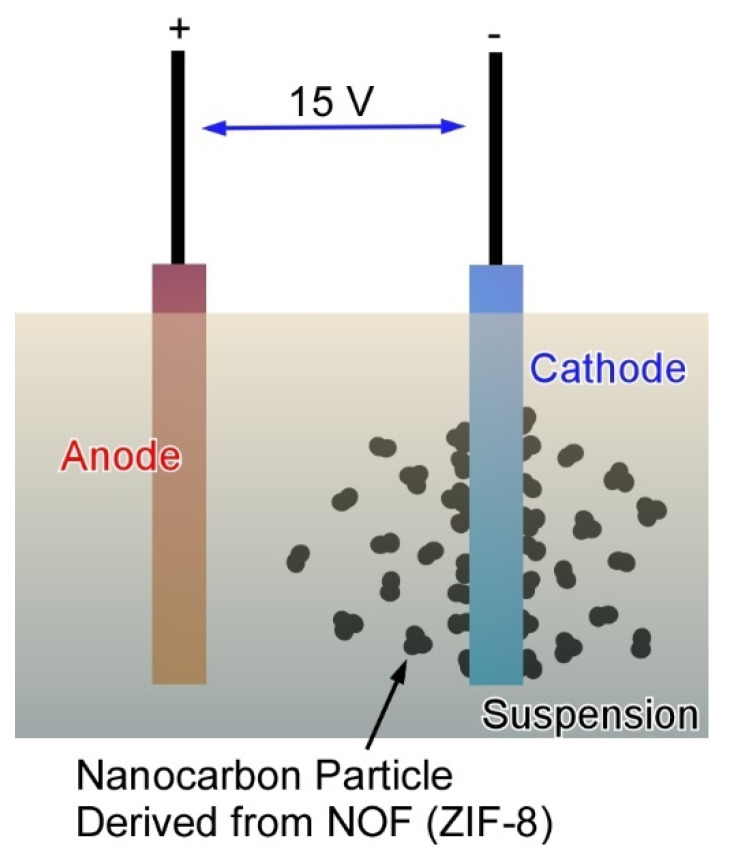
Fabrication of cathode electrode structures for flexible micro-supercapacitors through electrophoresis method nanocarbon materials prepared from ZIF-8 particles.

In fields in micro-electromechanical systems (MEMS), portable micro-supercapacitors would have high usability because of their cyclability and high power density. The nanoarchitected nanocarbon materials are further integrated into flexible micro-supercapacitors as recently reported by Henzie and co-workers (Figure 19) [186]. In their approach, nanocarbon materials prepared from ZIF-8 particles were immobilized through a simple electrophoresis process onto the corresponding electrodes. The ZIF-8 particles were first carbonized into nanocarbon materials under a nitrogen atmosphere at 800 °C. The obtained nanocarbon materials were then dispersed in water with Mg(NO_3_)_2_. Supercapacitor electrodes were prepared through an electrophoresis process in the nanocarbon-including aqueous suspension. Integrated electrode structures can be simply fabricated by this electrophoresis method. The prepared flexible micro-supercapacitors would be promising candidates for miniaturized flexible power supply systems in future applications.

**Figure 20:**
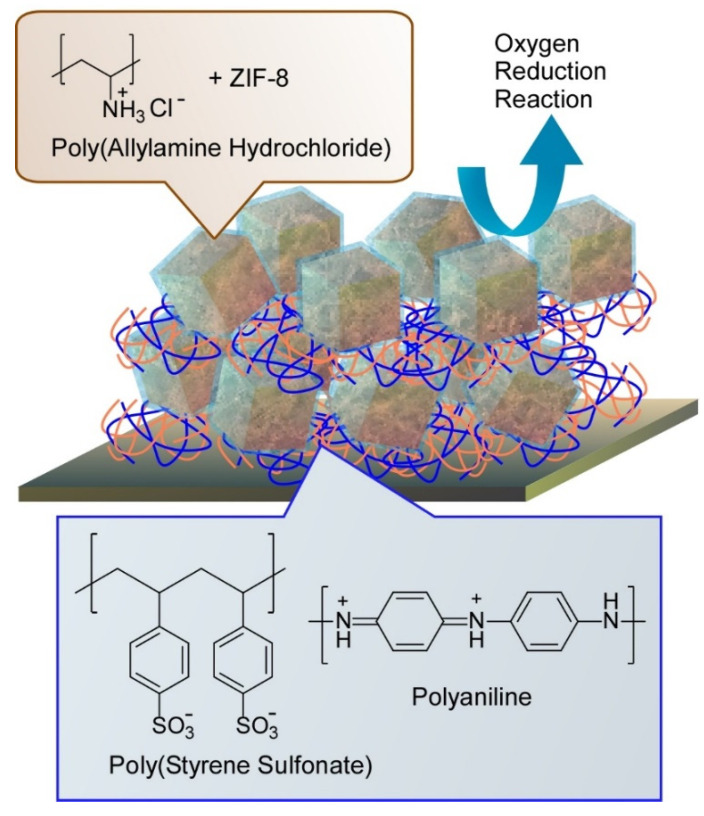
Nanoarchitectonics approach on the basis of layer-by-layer (LbL) assembly of conductive polymers and MOF complexes for better oxygen reduction reaction.

For fabrication of hierarchic structures for electrocatalysts in energy-oriented application, Azzaroni and co-workers reported a nanoarchitectonics approach on the basis of LbL assembly of conductive polymers and MOF complexes (Figure 20) [187]. For one of the key processes in energy converting electrochemical applications, materials to exhibit better oxygen reduction reaction have been actively explored. In their nanoarchitectonics approach, colloidal polymer suspensions of polyaniline/polystyrene sulfonate and MOF (ZIF-8) coated with polyallylamine hydrochloride were first prepared, and then they were alternately assembled into hierarchical layered structures. With the prepared nanoarchitectonics structures, catalytic performances for oxygen reduction reaction were enhanced through synergic effects of electrocatalytic properties of the conducting polymer and O_2_-absorbing MOF structures. Hierarchic construction of two different components with their own roles leads to better performances upon synergic functional coupling.

The abovementioned examples are only part of the huge number of approaches in the corresponding energy-orientated applications. However, these examples well elucidate necessary common features in these applications. Energy conversion functions requires several functional relays and the individual functional units have to be structurally well integrated. Therefore, better functional units have to be constructed into better structures for better energy performances. As shown above, nanoarchitectonics processes from nano-units to materials systems can satisfy these demands. The nanoarchitectonics strategies would play important roles in energy-related applications although this concept has been utilized in throughout of the corresponding research histories without being paid much attention.

### 3.2. Bio-Related Applications

Bio-related research results are undoubtedly important for social life activities including environmental monitoring [188,189], sensing [190,191], bio-remediations [192,193], drug delivery [194,195], therapies [196,197], and the other biomedical applications [198,199]. In addition, biomaterials such as amino acids, peptides, proteins, oligosaccharides, and nucleic acids are regarded as powerful components to form self-assembled structures in supramolecular chemistry and materials science [200,201,202]. Indeed, the hierarchical constructions of biological systems can be regarded as naturally occurring nanoarchitectonics systems where hierarchical assemblies of biomolecules result in incredibly high functional organizations such as living cells. Based on similarities between biological organizations and nanoarchitectonics-based structure-formations, bio-related applications would be the most important targets of nanoarchitectonics approaches.

**Figure 21:**
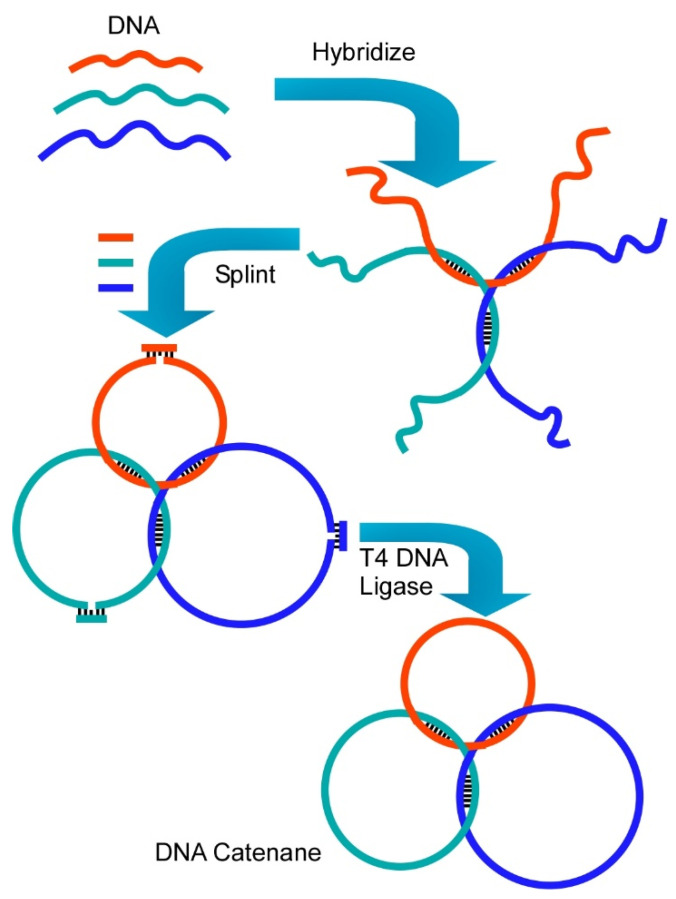
DNA Programmable nanoarchitectonics with DNA components to form advanced supramolecular structures such as interlocked structure, catenane.

Various bio-molecules have been used as components of nanoarchitectonics-based structural organizations. As summarized in a recent review article by Liang and co-workers [203], DNA and RNA can work as programmable nanoarchitectonics components to form advanced supramolecular structures such as interlocked structures and molecular machines (Figure 21). As interlocked nanoarchitectures, catenanes, rotaxanes, and their connectors can be constructed through specific complementary base-pairing of programmed DNA and RNA. Further nanoarchitectonics processes of these parts lead to formation of machine-like structures with dynamic functions including molecular walkers, molecular transporters, molecular shuttles, nanorobots, nanopumps, molecular amplifiers, and molecular logic gates. Fundamental designs of DNA and RNA are based on linkages of (deoxy)ribose, and phosphate, and, nucleobases. The former two units are common within any DNA or RNA structures, and only sequential differences of four kinds of nucleobases (adenine, guanine, cytosine, and thymine (or uracil)) decides their structures and roles. It is amazing fact that such a simple design concept can create a huge variety of molecular machines. As seen in these examples, biomolecules probably have optimized structure designs for molecular organization through billions of years of natural evolution. Biomolecules are highly useful for molecule-to-material nanoarchitectonics [204,205].

**Figure 22:**
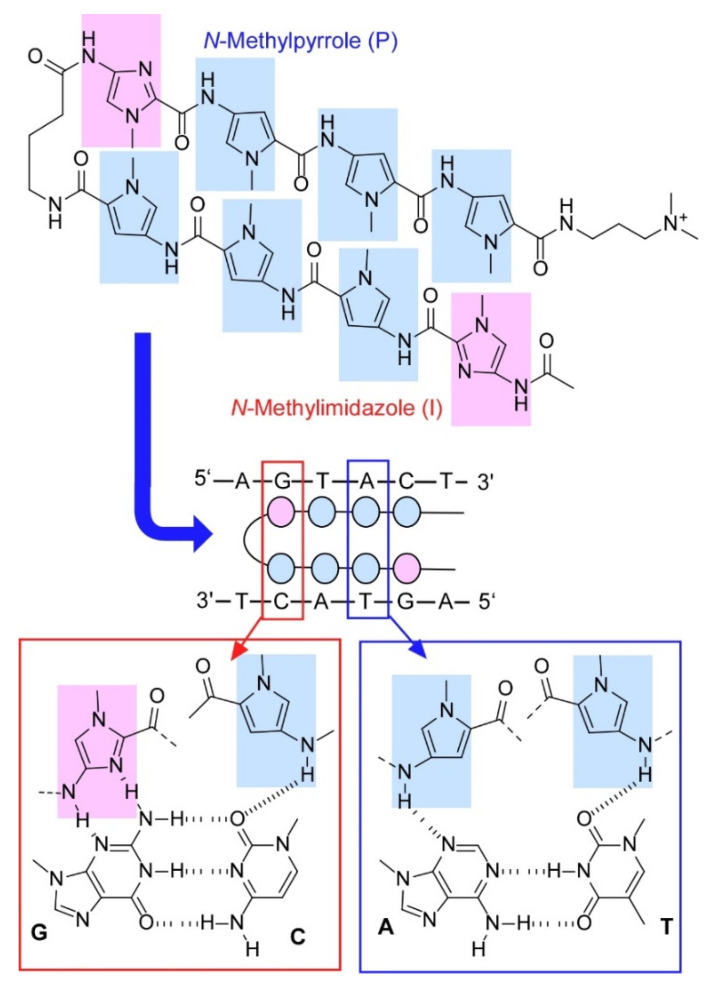
A polyamide with *N*-methylpyrrole (**P**) and *N*-methylimidazole (**I**) with bending hairpin structure binds to the minor groove of DNA upon hydrogen bond formation to specific nucleobases.

Exploration of artificial molecules that specifically interact with DNA would lead to various bio-related functions. For example, *N*-methylpyrrole (P) and *N*-methylimidazole (I) polyamides can be designed to act as sequence-specific DNA-binding ligands as described in a recent review article by Bando and Sugiyama [206]. The synthesized PI polyamides can bind to minor groove of double-stranded DNA and would have functions to regulate the specific gene expression or to visualize specific DNA sequences in living cells. One example of binding motif of PI polyamides to DNA is shown in Figure 22. The PI polyamides adopts bending hairpin structures through an aminobutyric acid spacer and binds to the minor groove of DNA upon hydrogen bond formation to specific nucleobases. Artificial gene switches can be nanoarchitected using the PI polyamide motifs for controlling expression of specific genes and further for binding better treatments for certain kinds of diseases. PI conjugate molecules capable of binding to target gene sequences are also developed as DNA imaging reagent. These molecular nanoarchitectonics with DNA binding capability would lead to development of specific gene-targeting drugs.

**Figure 23:**
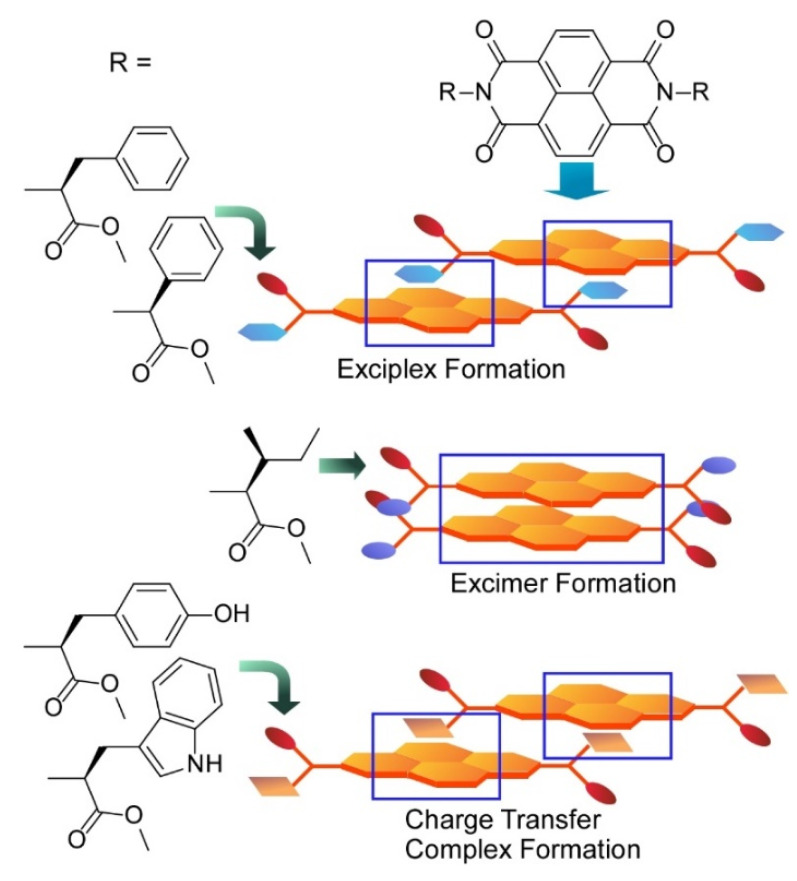
Assembling structures of naphthalenediimides conjugated with amino acid to form exciplex, excimer, and charge transfer complex depending on α-substituent of amino acids.

Roy and Govindaraju reported regulation of supramolecular assembly of arylenediimide derivatives with amino acid side groups [207]. Amino acid molecules have basic capability of forming hydrogen bonding, and their assembled motifs can be a widely modulated depending on side chain structures. As exemplified in Figure 23, assembling structures of naphthalenediimides conjugated with amino acid residues shifted with changes of α-substituents. Not limited to morphological controls in zero-, one-, two-, and three-dimensional structures, optical properties were also modulated through interaction of aromatic groups. Exciplex was preferentially formed in assembly of naphthalenediimides with phenyl substituents. Assembly of naphthalenediimides with isoleucine led to formation of excimers with specific zero-dimensional particles. Tyrosine and tryptophan substituents induced formation of charge transfer complex. Assembly-based nanoarchitectonics structures can be delicately modulated only with changes of tiny molecular structures. These molecular-based approaches are also called molecular architectonics.

**Figure 24:**
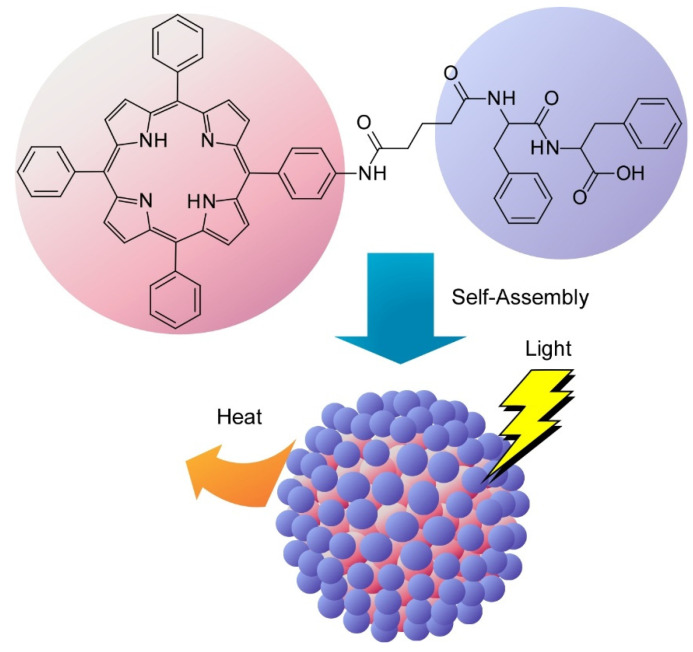
Photothermal nanodots from peptide-porphyrin conjugates for photothermal antitumor therapy.

Yan and co-workers are developing peptide-based nanoarchitectonics for bio-related applications [208]. Even and short peptides form various assembled structures, and these peptides also co-assemble with the other components. Therefore, variously shaped assembled materials with various components can be nanaoarchitected using short peptides. The obtained materials are utilized in may applications including phototherapy, biomimetic photosystems, and oriented microtubes for optical waveguiding. For example, they nanoarchitected photothermal nanodots from peptide-porphyrin conjugates for photothermal antitumor therapy (Figure 24) [209]. In the assembled nanodots, emission of fluorescence and production of singlet oxygen were efficiently suppressed upon strong π-stacking for highly efficient light-to-heat conversion process. The nanodots of the peptide-porphyrin conjugates are highly biocompatible and are capable of efficient tumour ablation. Flexibility, versatility, and adaptability of the peptide nanoarchitectonics approach can be extended to various systems toward versatile clinical translation.

Yan and co-workers include coordination-driven self-assembly of amino acids for tumour accumulation of curcumin with sufficient biological stability [210]. In this case, curcumin-including nanoagents were formed through self-assembly of amino acid derivative (9-fluorenylmethyloxycarbonyl-l-histidine) with the aid of coordination with Zn^2+^. The nanoarchitected nanoagents are capable of protecting curcumin from attack by hydroxide ions upon molecular stacking and metal coordination. The sizes of the curcumin-including nanoagents were kinetically thermodynamically controlled upon appropriate combinations of coordination and the other noncovalent interactions, which optimized antitumor therapy effects. In tumour-like environments, high loading capabilities of the drug and their responsible release properties were confirmed in addition to the enhanced stability. These features are advantageous for antitumor therapy. Because many antitumor drugs often bear metal-binding sites, this strategy can be applied to many targets. Nanoarchitectonics approaches to coordination-assisted self-assembly of biomolecules would be useful for antitumor therapy.

**Figure 25:**
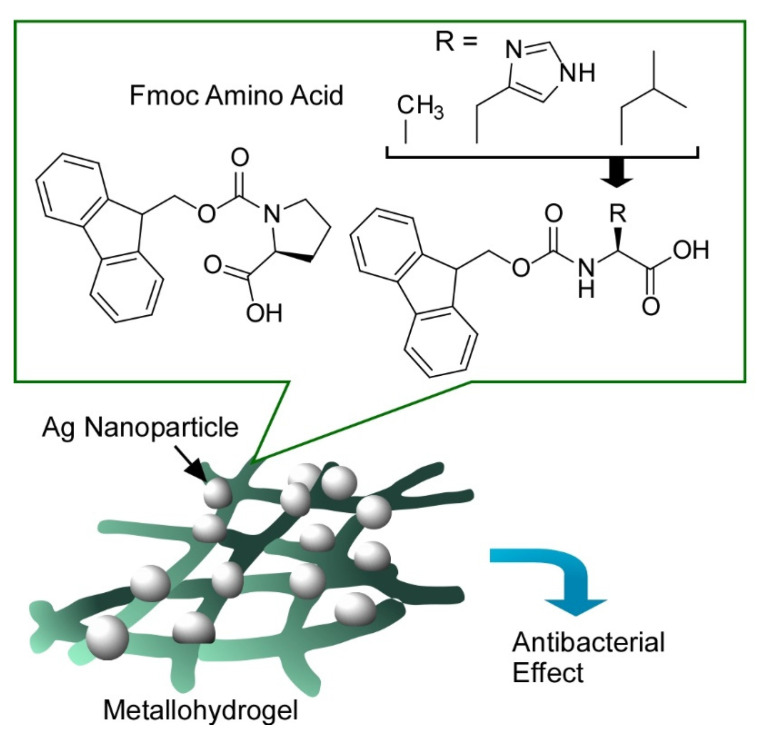
Metallohydrogels prepared from self-assembled nanofibers of Fmoc amino acids and Ag nanoparticles for antibacterial effects.

They also applied this strategy to preparation of antimicrobial biometallohydrogels by coordination with Ag ions [211]. Hydrogel nanofibers were prepared with Fmoc-protected amino acids such as alanine, histidine, leucine, and proline (Fmoc: 9-fluorenylmethyloxycarbonyl group). Based on coordination capability of the prepared hydrogel materials, Ag^+^ was immobilized, which was further converted into Ag nanoparticles upon local and mild mineralization (Figure 25). The finally nanoarchitected biometallohydrogels have several advantages in bio-related applications including sustained release, localized delivery, prolonging drug effect, and reduced drug dosage. At contact events of the biometallohydrogels with cells, Ag^+^ and Ag nanoparticles can directly interact with the cell surfaces of bacteria. Morphological changes of the cell walls were then induced accompanied with alteration of the permeability of the cell membrane. Detachment of the plasma membrane and subsequent leakage of the cytoplasm occurred, resulting in cell death. Antibacterial effects were significantly triggered for Gram-negative and Gram-positive (*Escherichia coli* and *Staphylococcus aureus*, respectively) bacteria in living cells and mice. As shown in this example again, coordinated self-assembly with amino acids, short peptides, nucleic acids, and metal ions is capable of nanoarchitecting biocompatible materials for various bio-related applications.

**Figure 26:**
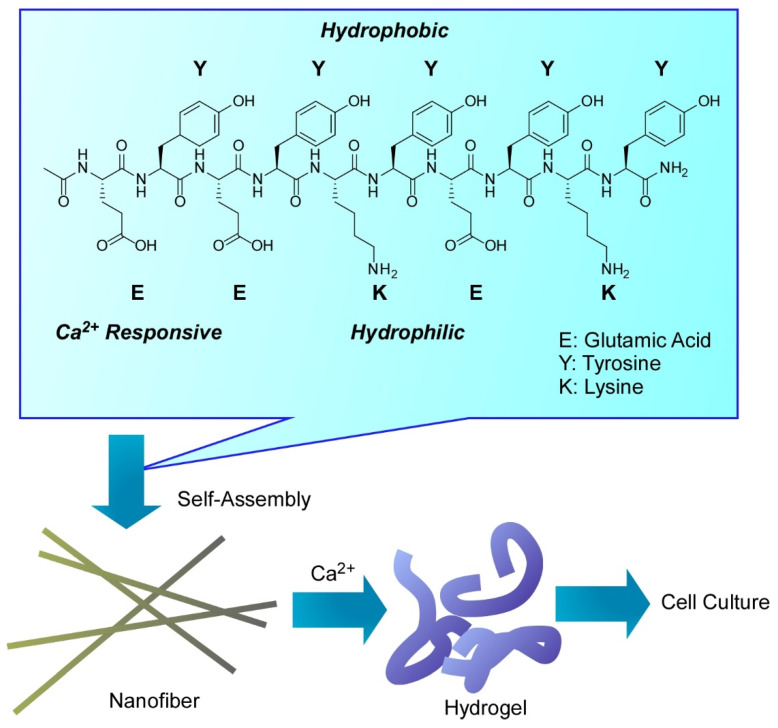
Formation of networked nanofibers and hydrogel with designed peptide (Ac-EYEYKYEYKY-NH_2_: E, glutamic acid; Y, tyrosine; K, lysine) with the aid of Ca^2+^.

Mihara and co-workers utilized well-designed oligopeptides for various biomedical applications [212]. The designed peptide (Ac-EYEYKYEYKY-NH_2_: E, glutamic acid; Y, tyrosine; K, lysine) formed networked nanofibers and hydrogel in the presence of Ca^2+^ (Figure 26). The designed sequence of alternate hydrophobic and hydrophilic residues is advantageous to form hydrophobic and hydrophilic sides within nanofibers with a β-sheet conformation. This amphiphilic nature of the basic assembling structure also assist further assembly to hierarchical structures. Glutamic acid at the *N*-terminal of the peptide sequence had a crucial role in responses to Ca^2+^. These nanoarchitected structures can work as mimics of natural extracellular matrices for drug delivery, cell culture, and tissue engineering applications. The formed hydrogels provided cell-compatible environment for cell adhesion, growth, and differentiation with freedoms of shape-forming. High drug-loading capability and shape-shifting nature of the hydrogel leads to fabrication of injectable materials for therapies of local delivery. In addition, construction of three-dimensional tissue and organ mimics would become possible with these features.

**Figure 27:**
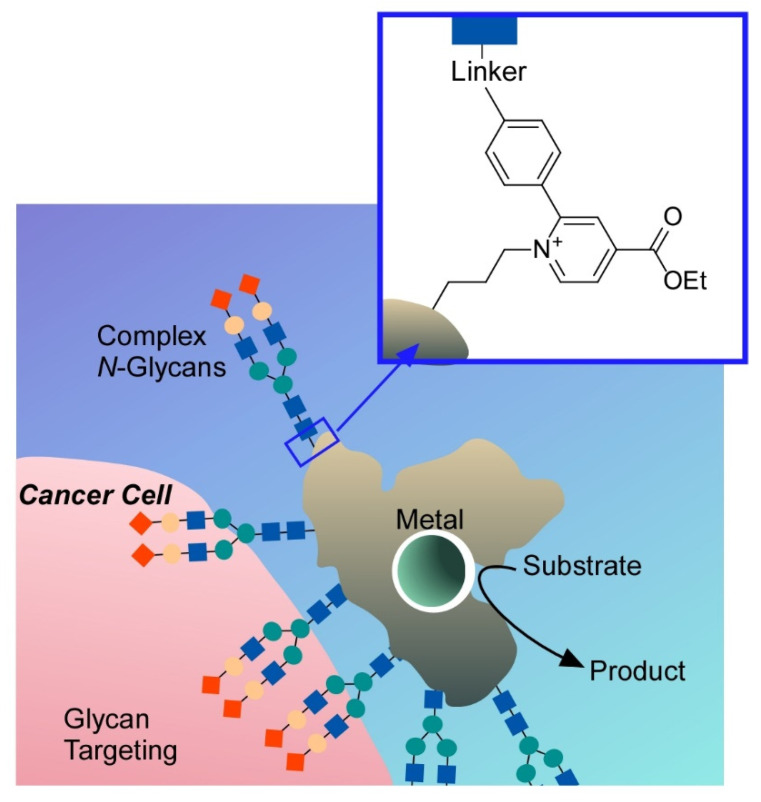
Nanoarchitectonics of artificial metalloenzyme on the basis of glycosylation of proteins.

As seen in naturally occurring systems, the ultimate peptide nanoarchitectonics goal would be the construction of protein-like and enzyme-like structures with functions. Materials conversion by enzymes is one of the sophisticated functional bio-actions with incredibly high specificity and efficiency working under ambient mild conditions [213,214]. Therefore, research efforts to develop artificial enzymes using supramolecular structures [215,216] and nanomaterials [217,218] have been continuously made. Instead of using non-biomaterials, modification of bio-originated materials based on their advanced intrinsic structures is also promising method to fabricate artificial enzymes. Tanaka and Vong summarized their approaches to nanoarchitect artificial metalloenzymes on the basis of glycosylation of proteins [219]. As depicted in Figure 27, introduction of glycan-dependent targeting modules and metallic biocatalytic sites to the original protein body produces glycosylated artificial metalloenzymes. This kind of nanoarchitectonics strategy on naturally occurring biomolecules is a promising way to construct highly functional systems.

The section above describes several examples of bio-related applications based on nanoarchitectonics approaches. Of course, these applications are only a limited selection from the huge variety of bio-related applications. However, essential features of bio-related nanoarchitectonics are included. Because biological functional systems are organized through self-assembly of molecular nano-units, bio-molecules and their mimic molecules must be good components for nanoarchitectonics from molecules to functional material systems. Bio-related applications would be promising and powerful targets for nanoarchitectonics research [220,221,222,223].

## 4. Perspectives

In this review article, the basics and some application examples of the nanoarchitectonics approaches are briefly explained. Some examples on atom/molecular-level nanoarchitectonics to create functional materials and related structures from atomic and molecular unites reveal the indispensable contributions of surface science and nanotechnology to organic chemistry. Although organic syntheses are thought to be well-established research fields, advanced nanoarchitectonics protocols can open new pages even in this classic science field. For further assembly of molecular units into materials, media for nanoarchitectonics become important. Interfacial environments are beneficial for fabrication of materials with nanostructure-based functions where molecular unit can be assembled anisotropically with certain orientations. Advanced applications such as energy conversion and antitumor therapies often require functional relays of individual components. Nanoarchitectonics fabrication of asymmetric and hierarchic organization with functional components is indispensable for material functions with better performances. Upon strong social demands, huge research efforts for social demands for energy [224,225,226], environment [227,228], and biomedical [229,230] issues are continuously made with promising results. Introduction of nanoarchitectonics into materials design and synthesis for these important demands would lead to further improvement and innovation of functional systems.

Despite various examples on nanoarchitectonics approaches in the preparation of functional materials systems at different scale regions, total constructions from simple molecules (or atoms) into complicated material organizations with sophisticated functions have not been accomplished well, even with nanoarchitectonics approaches. As compared with simple equilibrium self-assembly, the nanoarchitectonics strategies are supposed to be much more capable of organizing complicated functional structures with sufficient asymmetry and hierarchy. Hierarchical structural features from molecules to total materials systems are commonly observed in biological functional systems such as energy and signal conversion systems. Therefore, currently existing functional biosystems would be nice masterpiece specimens for successful nanoarchitectonics [231,232]. The nanoarchitectonics approaches have strong bio-similar features, and constructions of bio-like high functional systems would be one of the ultimate goals [233,234]. Sophisticated functions and structures in biological systems are the result of evolutionary processes over billions of years, but nanoarchitectonics have to be complete their task within a few decades [235]. Newly developed technologies such as machine learning and artificial intelligence [236,237,238,239] could assist the rapid evolution of molecular and materials nanoarchitectonics approaches. In addition, analytical sciences including advanced sensors could contribute to these research flows [240,241]. Especially, developments of novel analytical methods allow one to visualize the actual process and identify the products but so far their practical application for obtaining larger quantities of product still has to be elaborated.
